# Immune Cells and Immunoglobulin Expression in the Mammary Gland Tumors of Dog

**DOI:** 10.3390/ani11051189

**Published:** 2021-04-21

**Authors:** Alessandra Sfacteria, Ettore Napoli, Claudia Rifici, Daria Commisso, Giada Giambrone, Giuseppe Mazzullo, Gabriele Marino

**Affiliations:** Department of Veterinary Sciences, University of Messina, 98168 Messina, Italy; enapoli@unime.it (E.N.); claudiarifici35@gmail.com (C.R.); dariaep@libero.it (D.C.); ggiambrone97@gmail.com (G.G.); gmazzull@unime.it (G.M.); marinog@unime.it (G.M.)

**Keywords:** neoplasm, canine, macrophages, mast cells, B-cells

## Abstract

**Simple Summary:**

Among products released by inflammatory cells in the tumor microenvironment, immunoglobulins have been implicated in the pathogenesis and progression of various types of human cancer, but their role in veterinary oncology remains to be fully investigated. This paper aims to describe some of the immune cells inhabiting the tumoral microenvironment and their relationship, if any, with the expression of immunoglobulins. Simple carcinomas of the canine mammary gland showed the highest number of macrophages and B-cells in the so-called invasion areas, along with a strong epithelial immunofluorescence for IgA and IgG. Understanding the crosstalk between the tumor -associated immune cells and the neoplastic epithelium could be pivotal to unravel the pathways that lead to tumor regression or, conversely, promote tumor progression and resistance to chemotherapeutic drugs.

**Abstract:**

Inflammatory cells have a role in tumor progression and have prognostic and therapeutic potential. The immunohistochemical expression for Mast Cell Tryptase, Macrophage Marker, CD79a, IgA, IgM and IgG on 43 cases of canine mammary gland lesions was analyzed. In hyperplasia, a few B cells (BCs) and Tumor-Associated Macrophages (TAMs) were observed, while the number of Tumor-Associated Mast Cells (TAMCs) was the highest. In the peritumoral stroma of malignant lesions, low number of TAMCs and a high number of TAMAs and BCs were present. Immune cells of each type were always lower in the intratumoral than peritumoral stroma. Positivity to CD79a was also detected in the epithelial cells of simple and micropapillay carcinomas. Immunoglobulin reactivity was mainly located in the epithelial cells where an intense positivity to IgA and IgG and a weak positivity for IgM were detectable. On the basis of our preliminary results and literature data, we suggest that such cells and molecules could be directly involved in the biology of canine mammary gland tumors. In breast cancer, stromal inflammatory cells and cancer derived immunoglobulins have been correlated with the progression, malignancy and poor prognosis of the tumor. The results herein reported show that the dog’s mammary gland epithelium also expresses immunoglobulins, and they mostly show a direct relationship with the infiltration of macrophages. In addition, this study shows that the infiltration of mast cells, B-cells and macrophages varies depending on the degree of malignancy of neoplasia.

## 1. Introduction

The tumor-promoting inflammatory environment is one of the major cancer hallmarks, and how it influences tumor progression—and metastasis—or regression is raising great interest [1]. Cancer has been compared to a “wound that does not heal”, based on the observation that the tumor microenvironment is comparable to a state of chronic inflammation [2]. Indeed, immune cells, such as macrophages and mast cells, release soluble agents, such as cytokines and chemokines, that contribute to the tumor fate. Tumor-associated macrophages (TAMs) are present in all the stages of tumor progression. TAMs are endowed with remarkable plasticity and, according to the environmental stimuli, can ignite inflammation or exert anti-inflammatory and immunosuppressive reactions [3,4]. Tumor-associated mast cells (TAMCs) have been described along the invasion edges of malignant tumors. They are able to release molecules that promote tumor growth both as preformed mediators stored in granules and as ex novo synthesized mediators. It is not excluded, however, that such mediators as TAMC could also have anti-cancer effects. For example, TNF has a cytotoxic effect on cancer cells, while heparin and histamine protect against tumorigenesis [5]. Mast cells can behave, therefore, as dangerous promoters or innocent bystanders, depending on the characteristics of the microenvironment and their localization within the tumor [6]. The role of tumor-infiltrating B-cells is still debated and less investigated [7]. Similarly to other immune cells, some studies suggest that tumor infiltrating B-cells (Bc) have a protective role against disease progression by modulating the innate and/or adaptive immune system responses that results in increased tumor cell death [1]. It has been suggested that B-cells can promote carcinogenesis and that their presence is associated with increased tumor aggressiveness and poor prognosis in patients with solid tumors [8]. Immunoglobulins (Ig) are traditionally considered as an exclusive product of B cells. These molecules recognize and neutralize pathogens, or “non-self” cells, playing a crucial role in the regulation of the immune system mechanisms. It is noteworthy that in the last decades it has been demonstrated that many normal and pathological “non B”-cells, including podocytes [9], gametes [10], neurons [11] and endothelial cells [12] may also express immunoglobulins. Several scientific evidence underline the correlation between the expression of IgA, IgG and IgM and the tumor progression [13,14]. The aim of the study was (i) demonstrate that dog mammary gland lesions express immunoglobulins, and (ii) to correlate the TAM, TAMC and Bc numbers to different types of malignant tumors. An additional aim was to correlate immune cell type and presence to the grading of malignant lesions.

## 2. Materials and Methods

### 2.1. Animals, Tissue Processing and Histopathology

Forty-three mammary gland epithelial lesions, classified and graded in accordance with the published guidelines [15,16] were selected from the archives of the Unit of Veterinary Pathology, Department of Veterinary Sciences, University of Messina. Here is the detailed information on different dog mammary gland tumors that were selected: tubular/tubulopapillary carcinoma (10 cases), solid carcinoma (5 cases) and micropapillary carcinoma (8 cases). Each selected case was assigned a histologic degree from G1 to G3. Moreover, areas of hyperplasia/dysplasia (10 cases) were included in the study (Table 1). Data were reviewed on the basis of obtaining a consensus opinion of two pathologists (A.S., G.Mar.). The phenotypic characterization of TAMC, TAM and Bc was performed by immunohistochemistry (IHC) for Tryptase, Macrophage Marker and CD79a. Slides of each sample underwent direct immunofluorescence (IF) for immunoglobulins (the primary Abs are detailed in Table 2). Additionally, a double IF was performed for anti-cytokeratin (CK) and anti-IgG antibodies to check for colocalization of immunoglobulin G and the epithelium.

### 2.2. Immunohistochemistry and Immunofluorescence

Five micrometer slices were steamed in 0.01 mol/L sodium citrate buffer, pH 6, in a microwave oven for 15 min. Endogenous peroxidase activity was quenched by 0.3% hydrogen peroxide in methanol, while aspecific protein reactions were blocked by incubation with 2.5% BSA for 30 min.

For immunofluorescence, an additional bath of 10 min in 0.1% sodium borohydride (Merck, KGaA, Darmstadt, Germany), to reduce autofluorescence, was performed. Slides were after incubated over night at 4 °C with primary FITC conjugated Abs followed by rinsing in PBS and cover slipping for IF (Vectashield anti fade mounting medium with dapi, Vector Laboratories, Inc., Burlingame, CA, USA) or an incubation at room temperature with a biotinylated goat anti-mouse (BIOSPA, SPA Società Prodotti Antibiotici, Milan, Italy) or peroxidase conjugated anti-mouse (Santa Cruz Biotechnology, Dallas, TX, USA) secondary Ab for IHC or TRconjugated goat anti-mouse secondary Ab for double IF (Santa Cruz Biotechnology, Dallas, TX, USA).

An additional reaction was carried out by an avidin-peroxidase complex for biotinylated secondary antibody (BIOSPA, SPA Società Prodotti Antibiotici, Milan, Italy).

All the immunohistochemical reactions were developed with Vector Nova Red (Vector Laboratories, Inc., Burlingame, CA, USA) and counterstained with hematoxylin.

For each sample negative control was also performed by omission of primary Ab or substitution with normal immunoglobulins from the same species of primary Abs. Immunohistochemical stains were interpreted by assessing the cytoplasmic and/or membrane immunoreactivity.

The slides, prepared as above, were observed under an optical microscope (DMI6000, Leica Microsystems) connected to a camera and image analysis software (Leica Application Suite X, Leica Microsystems), and a numerical count of stained immune cells was performed.

In the cases of hyperplasia/dysplasia, cells in the intralobular and perilobular stroma were considered, while in the neoplastic lesions 10 intratumoral and 10 peritumoral microscopic fields were observed at 40x magnification and the numerical count of TAMC, TAMS and Bc was performed.

### 2.3. Statistical Analysis

Data on the numerical count were reported on spreadsheet using Microsoft Excel for Mac (16.44), and once assessed the normal distribution of the dataset using the Kolmogorov-Smirnov test of normality, Student t-test was performed to investigate the potential differences in the presence of Mast Cells, Macrophages and B-cells in intratumoral and peritumoral area, and between benign and malignant lesions.

One-way analysis of variance (One-way ANOVA) was carried out to compare the number of Mast Cells, Macrophages and B-cells among the different malignant histotypes.

Pearson’s test was performed to assess any correlation between the different immune cells’ number and the grading of malignant lesions.

All the statistical analysis was performed using the software GraphPad Prism version 5.1 (GraphPad Software, San Diego, CA, USA). A *p*-value < 0.05 was considered statistically significant.

## 3. Results

### 3.1. Hyperplasia/Dysplasia

**Figure 1:**
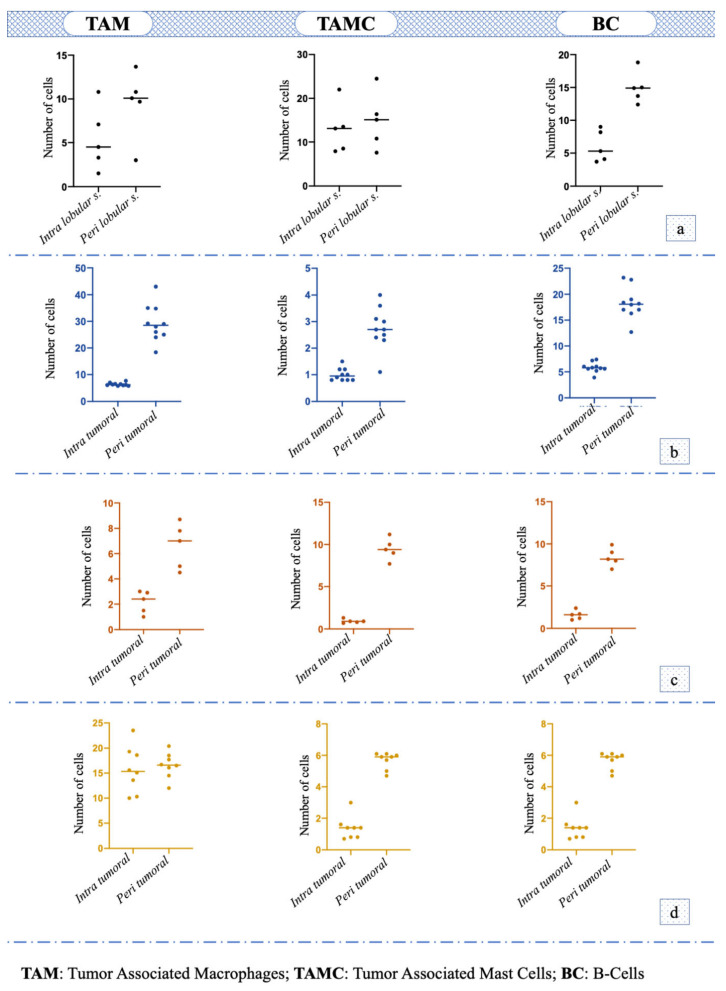
Number and distribution of tumor-associated macrophages (TAM), tumor-associated mast cells (TAMC) and B-cells (BC) in: (**a**) hyperplasia/dysplasia; (**b**) tubular or tubulopapillary carcinoma; (**c**) solid carcinoma; (**d**) micropapillary carcinoma.

Immunohistochemical staining for Macrophage Markers showed small groups of positive cells in the stroma between lobules of the hyperplastic areas (5.44 ± 3.24) and a numerical increase in the surrounding stroma (9.46 ± 3.53) any statistical difference was observed (*p* = 0.1316); the staining for Mast Cell Tryptase antibody showed that TAMC number within the lobules was 13 ± 5.5 and that in the surrounding stroma was 14.88 ± 5.73, which were almost similar (*p* = 0.6362). The immunohistochemistry for the CD79a marker revealed that the number of Bc in the stroma between lobules was significantly lower (6.06 ± 2.15) compared to that of the surrounding stroma (14.96 ± 8.13) (*p* = 0.0079) (Figure 1a).

### 3.2. Tubular/Tubulopapillary Carcinoma

**Figure 2:**
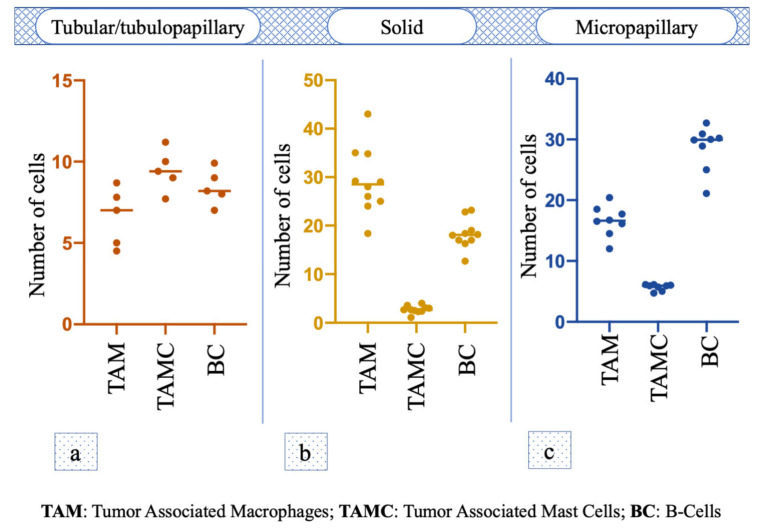
One-way analysis of variance (One-way ANOVA) of TAM, TAMC and BC number among the different malignant histotypes. (**a**–**c**) Tumor associated mastocytes in the footnotes has been replaced by Tumor Associated Mast Cells.

Immunohistochemical staining for Macrophage Marker showed that in the intra tumoral stroma the number of TAM was significantly lower (i.e., 6.38 ± 0.55) compared to the peritumoral stroma (29.24 ± 6.50; *p* < 0.0001); the staining for Mast Cell Tryptase antibody showed that the TAMCs’ number in the intratumoral stroma (1 ± 0.22) was significantly lower (*p* = 0.0006) compared to peritumoral area (2.74 ± 0.74). Additionally, the number of Bc was significantly lower in the intratumoral area (5.84 ± 0.93) compared to the surrounding stroma (18.36 ± 2.89; *p* < 0.0001) (Figure 1b). TAM was the immune cell that was most recorded (*p* < 0.001; F = 179.9) in this histotype (Figure 2a).

### 3.3. Solid Carcinoma

Macrophage Marker showed that in the intratumoral stroma, the number of TAM was significantly lower (i.e., 2.16 ± 0.78; *p* = 0.0028) compared to the peritumoral stroma (6.6 ±1.61); the TAMC number was significantly lower (*p* < 0.0001) in the intratumoral microenvironment (0.92 ± 0.20) compared to peritumoral one (9.46 ± 1.15). The number of Bc was significantly lower in intratumoral area (1.58 ± 0.48) compared to the surrounding stroma (8.42 ± 0.97; *p* < 0.0001) (Figure 1c). No statistical difference was observed among the number of different cells (*p* = 0.07, F = 3.845; Figure 2b).

### 3.4. Micropapillary Carcinoma

The number of TAM in the intratumoral stroma (15.75 ± 4.30) and peritumoral stroma (16.55 ± 2.37) was almost the same, and no statistical difference was observed (*p* = 0.6601). The TAMC number was significantly lower (*p* = 0.0002) in the intratumoral area (1.38 ± 0.69) compared to peritumoral one (5.67 ± 0.50), while the number of Bc was also significantly lower in the intratumoral microenvironment (12.82 ± 1.41) compared to the surrounding stroma (28.4 ± 3.70; *p* = 0.0002) (Figure 1d); in particular, Bc was the immune cell mostly present in this histotype (*p* < 0.0001, F = 198.9; Figure 2c).

### 3.5. Correlation between the Different Immune Cells’ Numbers and the Grading of Malignant Lesions

A positive correlation between the overall number of TAM and the grading was observed (R^2^ = 0.330, *p* = 0.0041), while no correlation was observed between the overall TAMC (R^2^ = 0.140, *p* = 0.0778) and Bc (R^2^ = 0.006, *p* = 0.7162) number and the grading. Regarding the intratumoral environment, no correlation was observed between the TAMC, TAM and Bc number and the histological grading; by contrast, the numbers of TAMC (R^2^ = 0.3390, *p* = 0.0036) and TAM (R^2^ = 0.3079, *p* = 0.0060) were positively correlated with the grading.

### 3.6. Epithelial Positivity for CD79a

Many epithelial cells showed immunohistochemical positivity for CD79a as follows: weak positivity for CD79a in hyperplastic areas of the mammary gland epithelium; strong and diffused positivity to all the layers of epithelial cells in tubulopapillary and micropapillary carcinomas.

**Figure 3:**
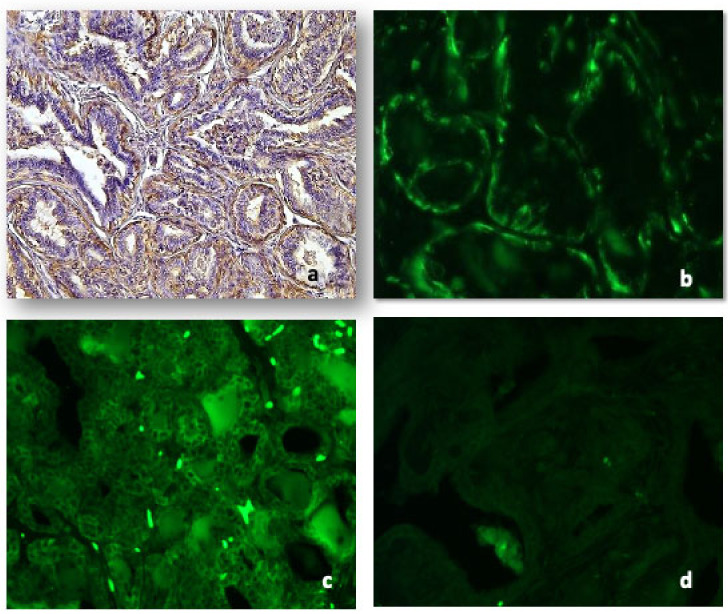
Representative images for: CD79a in tubular carcinoma both at IHC ((**a**), 10×) and IF ((**b**), 20×). Immunoglobulin A was detected both in the epithelium and stromal cells of tubular carcinoma ((**c**), 10×); immunoglobulin M was absent or weak ((**d**), 10×).

The positivity for Cd79a antibody, either on immunohistochemistry (Figure 3a) or immunofluorescence (Figure 3b), was identical.

### 3.7. Immunoglobulins

Anti-IgA antibody immunofluorescence was diffused in the epithelium of hyperplasia, confined to small groups of cells in solid carcinomas, more or less marked in intraluminal papillae of papillary tubular carcinoma and intensely marked in the micropapillary areas. Several stromal cells were positive both in benign lesions and malignant tumors (Figure 3c).

Immunofluorescence with anti-IgM antibodies was mild or absent in the various neoplastic histotypes considered. No positive stromal cells both in benign lesions and malignant tumors were detectable (Figure 3d).

**Figure 4:**
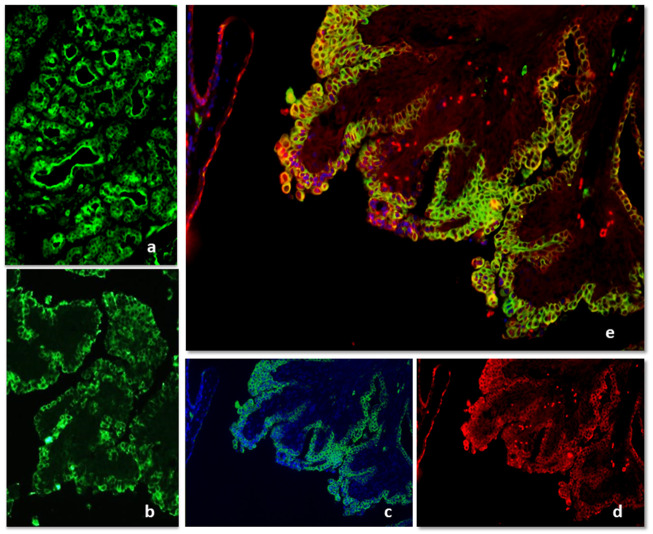
IF for Immunoglobulin G positivity in hyperplasia/dysplasia (**a**) and tubulopapillary carcinoma (**b**). The merging of IgG and CK immunofluorescence showed that in malignancies, IgG was mainly expressed by epithelial cells and that very few stromal cells were positive (**c**–**e**).

Immunofluorescence with anti-IgG antibodies appeared strong and localized in luminal cells in the hyperplastic/dysplastic lesions (Figure 4a). In malignant histotypes, the positivity was strong and diffused to the whole cell cytoplasm and all the cell layers in tubular or tubulopapillary carcinoma or localized in small nests and cords of cells in solid carcinomas (Figure 4b). The epithelial intensity decreased in typical intraluminal papillae of micropapillary carcinoma. The colocalization with the CK positivity showed that in malignancies IgG was mainly expressed by epithelial cells and that very few stromal cells were positive (Figure 4c–e).

## 4. Discussion

Immune cells can suppress or support the tumor initiation, progression and metastases. Cancer, on the other side, modulates the antitumoral response by secreting soluble mediators and interfering with the mechanisms of immunomodulation by activation or inhibition of immune cells. TAMCs have been widely demonstrated in various types of malignant tumors, such as breast carcinoma. They are activated either by direct contact or by other mechanisms such as the release of pro-tumoral mediators by neoplastic cells, release of mediators contained in the secretory granules of TAMC itself, adverse microenvironmental conditions such as hypoxia, presence of other activating molecules produced by the cells of the immune system, including light chains of immunoglobulins [17]. In veterinary medicine, the few data so far published have failed to draw attention to the critical role of these stromal cells in mammary gland tumors. It has been suggested that mast cell activation promotes angiogenesis, a crucial event for tumor progression and unfavorable prognosis [18]. It has been shown an increase in the number and distribution of Epor and Tryptase-positive MCs in the peritumoral stroma both in the normal/hyperplastic and neoplastic canine mammary glands tumors [19] and that tryptase positive MCs in canine mammary tumors are directly involved in the activation of metalloproteases, thus promoting the degradation of the extracellular matrix as well as the proliferation of vascular endothelial cells and cell differentiation [20]. Recently, it has been postulated that the low stromal density of toluidine blue TAMCs is predictive of a poor outcome for dogs bearing malignant CMGTs [21]. In the present paper, low tryptase-positive TAMCs in intratumoral stroma positively correlate with the grading. In addition to these data, we confirmed the higher number of tryptase-positive TAMCs in the hyperplastic areas of the mammary gland and in the stroma surrounding the cancerous growth, regardless of histological grading or histotype except for a slight increase in micropapillary carcinoma. These data, although not statistically significant, reveal the constant presence of TAMCs in precancerous lesions and in the so-called tumor invasion areas. A meta-analysis study on human solid cancer, including breast cancer, revealed that tryptase-positive TAMCs infiltration was significantly associated with decreased overall survival and disease-free survival [22]. Moreover, in our study, it was also observed that the cell number was directly related to the expression of IgG in tumor epithelial cells. Specifically, mammary tumors showed an increase of IgG in the carcinomatous areas which is contrary to what was observed for mast cell numbers. This behavior deserves further study. Mast cells, mostly known as IgE-dependent allergic reaction initiators, like other myeloid cells, also express receptors for the Fc portion of IgG antibodies and it would appear that IgG, if present in high quantities, is capable of binding to mast cells instead of IgE preventing their degranulation. In this view, mast cells can then be enrolled in a variety of adaptive immune responses and their activation and biological response can be adjusted, either positively or negatively, by antibody modulation [23]. Data on the FLC (immunoglobulin free light chains) expression, in preclinical models of tumor and their associated MCs, demonstrated that FLC activate MCs to produce molecules that enhance the tumor growth and that increased expression of FLC is indicative of poor survival [24]. The role of TAMs can vary, and tumor inhibition or progression may be dictated by their phenotype and activation pathway. Macrophages can be “educated” by the tumor microenvironment to stimulate angiogenesis, neoplastic progression and metastases [25]. In canine mammary carcinoma, it has been demonstrated that TAMs secrete canonical Wnt inhibitors that decrease tumor proliferation and development or induce the non-canonical Wnt pathway responsible of tumor metastasis [3]. In dogs, the high density of TAMs in malignant mammary tumors has been associated with poor survival and therefore considered of prognostic value [26,27]. Recently, it has been stated that benign mammary tumors display low macrophage counts when compared with malignant mammary tumors such as micropapillary carcinomas, solid carcinomas and carcinosarcomas [28]. Similarly, in breast cancer, the number of TAMCs and TAMs is higher in G2/G3 samples and has been correlated with the microvasculature density [29]. In agreement with the literature data, we found small groups of positive cells in the hyperplastic areas of the mammary gland, whereas an increase in invasive peritumoral areas and a statistically significant increase in the G1 histological grade of malignancy was assessed. With respect to intratumoral stroma, an increase of macrophages was observed compared to hyperplastic areas, especially in the most malignant histotypes, even though it was not possible to make a correlation with prognosis and survival due to lack of data. On the other hand, it has been widely assessed that tumor grade can be an affordable indicator of survival time in dogs affected by mammary gland cancer [30]. With regard to B-cell lineage, recently it has been assessed, both in mouse models and in humans, that B cells can support carcinogenesis by the modulation of innate and/or adaptive immune responses or enhancing the upregulation of pro-angiogenic genes [8] Antibody production and subsequent deposition of immune-complexes in tumor tissue is another mechanism whereby B cells may promote inflammation and neoplastic progression. B cells may also facilitate tumorigenesis. It has been demonstrated that immunoglobulins are also a product of proliferating epithelial cells in hyperplasia of mammary glands and normal epithelium close to cancer tissue [31]. Moreover, patients bearing cancers such as those originating from the breast epithelium show monoclonal gammopathy and elevated level of serum IgG, IgA, or IgM antibodies [32]. Tumor-derived IgG is likely to promote the survival and growth of epithelial tumor cells in an autocrine/paracrine fashion. Some authors have hypothesized that cancer-derived Ig could compete with B cell-derived Ig for the FcR on effector cells, thus inhibiting ADCC and help tumor immune escape [33]. A correlation between IgG expression and histologic subtypes of carcinoma showed that poorly differentiated breast cancer cells express more IgG than well differentiated ones [34]. Other in vivo evidence demonstrated that IgG positive metastases of breast tumors are related with a shorter disease-free interval from the time of mastectomy [31]. In vitro experimental assay demonstrated that B-cell infiltration and antibody production precede premalignant transformation and enhance tumor proliferation [35]. Recently, an epithelial immune transition (EIT) was proposed to explain the immune-suppressive micro-environment created by cancer cells including the expression of Ig motifs in cell surface molecules [36]. CD79a is a transmembrane protein, which, with CD79b, forms a heterodimer which constitutes the transduction fraction of the BCR (B-Cell receptor) signal after binding with IgA, IgM, IgD and IgE immunoglobulins and it is expressed in the very early stages of development of B cells, up to the last stage of maturation, including differentiation into plasma cells. In the veterinary literature, Perez et al. described CD79/IgA/IgM positive cell infiltrates in the microenvironment of regressing Canine Transmissible Venereal Tumor (CTVT), whereas CD79/IgG positive cells where increased in the progressing phase (Perez et al., 1998). Other works have identified B cells (CD79a and CD20 positive) in feline oral squamous cell carcinoma [37]. In the canine mammary gland tumor, B cell infiltration (CD20 positive) was not correlated with tumor malignancy [38]. In the literature, we found a single work in which a colocalization between IgM and CD79 had been demonstrated in human neoplastic epithelial cells [39]. Anyway, even if the immunoreactivity seems to be genuine, a cross-reactivity of the anti-cd79a Ab used in this paper with similar antigenic structures involved in immunoglobulin trafficking cannot be excluded. In conclusion, as previously described in human breast malignancies, our data describe the immunopositivity for IgG, above all, in some selected tumor histotypes, as well as the positivity for CD79 and their relationship with immune cell infiltrates. The low number of Ig positive cells in the tumor-associated stroma, despite the presence of CD79a positive cells, suggests that precursors of plasm cells could be involved in microenvironment biology but that the production of Igs may happen elsewhere in the tumor and that Igs could have a different role besides immunosurveillance even in the canine mammary gland. Moreover, among the plethora of immune cells that habit the tumor microenvironment, we could not assess which immune cell type was Ig-positive. On the other hand, the merging of the double IF for CK and IgG revealed a strong colocalization in many epithelial areas where the stroma was devoid of IgG positive immune cells. Further studies are running to better investigate the Ig site of production and the relation to proliferation, apoptosis, grading or survival rate after mastectomy. Moreover, it could be of overwhelming importance to understand the crosstalk between the tumor-associated immune cells with a view to enhancing the pathways that lead to tumor regression and, conversely, suppress the main mechanisms of tumor progression and resistance to chemotherapeutic drugs.

## 5. Conclusions

In conclusion, the findings of this preliminary morphological study add insight to the knowledge that the inflammatory tumor microenvironment modifies according to the tumor histotype and grading. Moreover, for the first time IgA, IgG, IgM and CD79a expression is demonstrated in the neoplastic mammary gland epithelium of dog.

## Figures and Tables

**Table 1:** Case series included in the paper.

Case #	Breed	Age	Sex	Histotype	Grade
1	Sicilian hound	7	F	Tubular Carcinoma	G1
2	Crossbreed	n.a.	F	Tubular Carcinoma	G1
3	Crossbreed	12	F	Tubular Carcinoma	G1
4	Yorkshire terrier	7	F	Tubulopapillary Carcinoma	G1
5	Crossbreed	5	F	Tubulopapillary Carcinoma	G1
6	German Shepherd	11	F	Tubulopapillary Carcinoma	G1
7	Yorkshire terrier	10	F	Tubulopapillary Carcinoma	G1
8	Yorkshire terrier	4	F	Tubulopapillary Carcinoma	G2
9	Cocker	15	F	Tubulopapillary Carcinoma	G2
10	Cocker	10	F	Tubulopapillary Carcinoma	G2
11	Breton	8	F	Micropapillary Carcinoma	G2
12	Shih-Tzu	10	F	Micropapillary Carcinoma	G2
13	German Shepherd	5	F	Micropapillary Carcinoma	G2
14	Jack Russel	5	F	Micropapillary Carcinoma	G2
15	Jack Russel	7	F	Micropapillary Carcinoma	G2
16	German Shepherd	10	F	Micropapillary Carcinoma	G2
17	Yorkshire terrier	7	F	Micropapillary Carcinoma	G3
18	Dalmatian dog	8	F	Micropapillary Carcinoma	G3
19	Golden Retriver	8	F	Solid Carcinoma	G2
20	Crossbreed	10	F	Solid Carcinoma	G2
21	Crossbreed	14	F	Solid Carcinoma	G2
22	Crossbreed	12	F	Solid Carcinoma	G2
23	Crossbreed	8	F	Solid Carcinoma	G3
34	Pomeranian	13	F	Hyperplasia/Dysplasia	
35	Crossbreed	9	F	Hyperplasia/Dysplasia	
36	German Shepherd	n.a.	F	Hyperplasia/Dysplasia	
37	Crossbreed	6	F	Hyperplasia/Dysplasia	
38	Yorkshire terrier	5	F	Hyperplasia/Dysplasia	
39	Poodle	10	F	Hyperplasia/Dysplasia	
40	Crossbreed	n.a.	F	Lobular Hyperplasia with Atypia	
41	Siberian husky	10	F	Hyperplasia/Dysplasia	
42	Poodle	6	F	Lobular Hyperplasia with Atypia	
43	Siberian husky	8	F	Hyperplasia/Dysplasia	

# number; n.a. not available; F female.

**Table 2:** Primary antibodies used for IHC and IF.

Antibody	Clone	Brand	Specificity	Dilution
Mouse anti CD79a	HM47/A9	Novocastra HM47-A9	B-Cell receptor	1:50
Mouse anti Macrophage Marker	MAC387	Santa Cruz Biotech MAC 387	Macrophages	1:200
Mouse anti Mast cell Tryptase	10D11	Novocastra 10D11	Mast cells	1:100
Mouse anti Pan-cytokeratin	AE1/AE3	Santa Cruz Biotech AE1/AE3	Epithelium	1:200
Goat Anti Dog IgM:FITC	n/a	AbD Serotec	Immunoglobulin M	1:100
Sheep Anti Dog IgG:FITC	n/a	AbD Serotec	Immunoglobulin G	1:100
Mouse Anti Dog IgG:FITC	n/a	Sigma	Immunoglobulin G	1:100
Goat Anti Dog IgA:FITC	n/a	AbD Serotec	Immunoglobulin A	1:100

## Data Availability

The data presented in this study are available on request from the corresponding author.
